# Role of emotional creativity and achievement motivation on trauma symptoms among university students

**DOI:** 10.3389/fpsyg.2023.1203226

**Published:** 2023-11-13

**Authors:** Asanka Bulathwatta, Rekhani Lakshika

**Affiliations:** Department of Psychology, Faculty of Arts, University of Peradeniya, Peradeniya, Sri Lanka

**Keywords:** emotional creativity, achievement motivation, trauma, trauma symptoms, university students

## Abstract

The aim of this study was to understand the levels of trauma symptoms and the mediating role of achievement motivation, along with emotional creativity, among university students. Many students from various faculties exhibit diverse levels of academic motivation due to their program structures. However, the traumatic experiences faced by students and the subsequent post-traumatic symptoms can lead to various psychosocial and emotional consequences, potentially impacting their emotional creativity and achievement motivation. The present study employed a quantitative approach, utilizing measures to assess emotional creativity, achievement motivation, and trauma symptoms within the sample. The sample under investigation comprised 337 undergraduates from nine different faculties at the University of Peradeniya, Sri Lanka. The study’s results indicated a significant correlation between emotional creativity and achievement motivation. While emotional novelty did not display a significant correlation with achievement motivation, emotional effectiveness and authenticity did show such a correlation. Notably, hyperarousal, as a trauma symptom, exhibited a negative correlation with achievement motivation. The study identified both direct and indirect impacts of emotional creativity on achievement motivation. The findings from the regression analysis suggested that the impact of emotional creativity on achievement motivation falls between higher and lower levels. Furthermore, the study concluded that trauma symptoms mediate the relationship between emotional creativity and achievement motivation among undergraduates.

## 1. Introduction

The world finds itself at a pivotal moment in human history, confronting an array of challenges on a daily basis. The global impact of the COVID-19 pandemic and the subsequent economic downturn have presented significant tests to international forums. Academics, too, have not been spared from these changes. The advent of online education, the emergence of blended teaching methods, and the transformations in academic environments have ushered in critical conditions and alterations in educational frameworks. Within this academic community, university students, as integral members, now confront a multitude of challenges. The exigencies of their circumstances often cast a shadow over their creativity and motivation. Notably, the rigors of these critical conditions frequently manifest in traumatic and depressive symptoms, adding further obstacles to their path. Moreover, the shift to distance learning and abrupt changes in the learning landscape intensify the array of challenges they grapple with each day. Initial investigations into the experiences of university students within the realm of traumatic events indicate that a significant portion, ranging from 45 to 84%, have encountered at least one traumatic event during the course of their lives (Bernat et al., 1998; Avant et al., 2010; Bachrach and Read, 2012; Grasso et al., 2012, as cited in Cantrell, 2016).

Sri Lankan university students, comprising a distinct group meticulously chosen through the University Grant Commission’s z-score-based selection process, are often regarded as the finest cohort among the nation’s school graduates. The University of Peradeniya, in particular, welcomes a substantial influx of undergraduates annually. In contemporary times, Sri Lanka is witnessing a heightened prevalence of psychosocial challenges within its university student population, surpassing previous levels. This phenomenon has arisen as a consequence of the ongoing economic recession. Its inception dates back to the early months of 2022, exerting a profound impact on various societal pillars, including educational institutions and the schooling system. Presently, Sri Lanka is grappling with acute deficiencies in foreign currency reserves. The nation finds itself in a state of default, facing challenges in servicing its foreign debts. This predicament has significantly contributed to the notable depreciation of the Sri Lankan rupee and has precipitated inflationary pressures (Mehta, 2022; Perera, 2022, as cited in Leanage and Saito, 2023).

A study conducted by the University of Colombo has identified academic workload, economic hardships, personal relationships, instances of ragging or collective student behavior, hostel life, and cultural shock or pressure as prominent factors that directly and subjectively contribute to the escalation of stress among undergraduates (Mahees, 2020).

As a result of the economic recession and the various personal challenges students encounter, numerous instances of psycho-social disturbances have been reported among students, prompting intervention from the university health center and relevant authorities. There has been a notable rise in suicidal ideation among students across all faculties. Tragically, certain cases of suicide have gained widespread attention through social media and popular publications.

Incidents of student violence, conflicts involving academic staff, and instances of aggression among undergraduates constitute a grave concern. Operating as a residential university, accommodating 60% of its student body, the university administration bears the responsibility of ensuring the psychological wellbeing of students, even within the hostel environment. Consequently, the university assumes a pivotal role as the primary institution accountable for the welfare of undergraduates. According to numerous academicians within the university, students exhibit diverse emotional patterns and variations in their academic motivation and achievements.

In today’s competitive landscape, individuals are constantly engaged in various forms of competition. Within the academic sphere, the focus centers on academic accomplishments and grades. However, the context shifts when it comes to universities, particularly for Sri Lankan university students. They must navigate a complex terrain encompassing numerous life challenges, cultural adjustments, and sub-cultural adaptation hurdles within the university, as well as contend with natural disasters, human-made crises, economic hardships, and an array of social issues. Table 1 displays various trauma symptoms that people experience, along with their nature and characteristics.

**TABLE 1:** Symptoms of trauma and their characteristics.

Trauma symptoms	Nature	Characteristics
Intrusive reactions	The ways the traumatic experience comes back to mind	Recurrent upsetting thoughts and images, strong emotional reactions to reminders of the experiences, and feeling that something terrible may happen again
Avoidance and withdrawal reactions	Avoiding people, places, and things that are reminders of the traumatic experiences, withdrawal reactions	Feeling of numb, detached engaged from others, losing interest in usual pleasurable activities
Hyper-arousal reactions	Sensitivity and anxiety to the event related matters	Symptoms may include sleep difficulties, difficulty concentrating, irritability, heightened jumpiness, nervousness, and a constant state of vigilance for potential danger

There exists a paucity of research addressing the domain of trauma symptoms among university students. Existing studies predominantly revolve around coping strategies within trauma, post-traumatic stress disorder, or post-traumatic growth frameworks. In certain instances, traumatic experiences have also been explored within the context of emotional intelligence and resilience. However, the interplay between emotional creativity and traumatic symptoms, and their collective impact on determining achievement motivation, remain underexplored and relatively scarce in the literature.

Research pertaining to trauma among students remains limited in scope. The majority of studies predominantly concentrate on the broader population or specific groups that have experienced particular forms of mistreatment or abrupt adversities in their lives. Stress and various emotional challenges encountered by university students are frequently addressed within numerous contexts of discussion.

Numerous studies have delved into the realm of academic performance and wellbeing among university students. However, there remains a dearth of research concerning traumatic experiences and the ensuing factors that aid individuals in surmounting the difficulties arising from such encounters. People often confront a plethora of challenges that impede their achievements and emotional wellbeing, all stemming from traumatic experiences.

**FIGURE 1:**
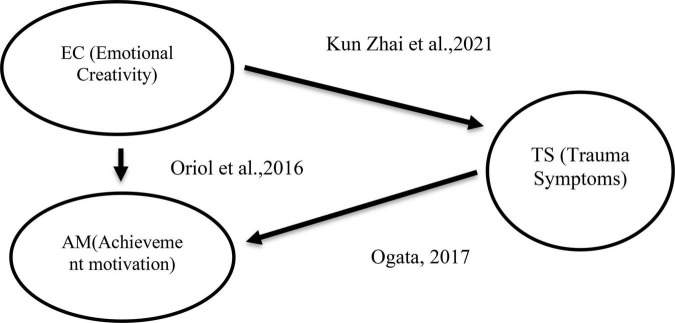
Model depicting relationships between variables.

When addressing traumatic conditions, Frazier et al. (2009) conducted a multisite study aimed at gauging the prevalence of exposure to traumatic events and the resultant symptoms among undergraduate students (*N* = 1,528) through online surveys. Their investigation revealed that a significant majority of students (85%) reported having undergone a traumatic event at some point in their lives, while 21% recounted such an experience occurring over a 2-month period during their university tenure. Their study illuminated a series of life-altering incidents that engendered trauma within the university student population. The event most frequently reported at both assessment time points was the unexpected demise of a loved one. Furthermore, experiences involving family violence, unwanted sexual attention, and sexual assault were linked to heightened levels of distress over the course of a lifetime. Notably, when designated as the most distressing event, sexual assault exhibited the strongest association with severe post-traumatic stress disorder symptoms. Events that evoked intense fear, helplessness, or horror, as well as those that were intentionally inflicted, were likewise correlated with elevated distress levels. Consistently, the total number of lifetime traumatic incidents demonstrated the most robust correlation with distress levels. Figure 1 depicts the model of the study, and it points out the causal relationship between the variables. This model was developed based on the previous studies referred in this research.

Bulathwatta et al. (2016) highlight a notable distinction in the emergence of traumatic symptoms stemming from natural trauma between German and Sri Lankan university students. The hierarchical regression analysis conducted in this study underscores that the influence of emotional intelligence on approach coping among Sri Lankan students is comparatively lesser when compared to its significance among German students. Additionally, the study reveals a recurrent pattern of resilience observed among German students. Conversely, Sri Lankan students tend to employ emotional intelligence and resilience as mechanisms to navigate and overcome traumatic experiences.

Emotional engagement holds significant importance within the framework of trauma. The emotional disturbances experienced by students exert a substantial influence on the determination of their coping strategies and academic productivity. Averill (1999), through the Emotional Creativity Scale, underscores the significance of emotional preparedness, novelty, and effectiveness as essential dimensions. Creativity, as delineated by Sternberg and Lubart, (1999, as cited in Alzoubi et al., 2021), can be viewed as an individual’s capacity to generate novel and valuable insights. However, in the context of traumatic symptoms among university students, there remains a notable absence of comprehensive exploration into the role of emotional creativity engagement.

The effects of traumatic symptoms on the academic environment and its accomplishments have received limited research attention. While traumatic stress symptoms can manifest in classroom behaviors, they often run the risk of being misinterpreted as issues related to attention or conduct (Goodman and West-Olatunji, 2010; Levine and Kline, 2007, as cited in Goodman et al., 2012).

Academic performance and creative abilities flourish when students experience happiness and emotional creativity (Ogata, 2017). Positive emotions serve as catalysts, motivating individuals to generate innovative ideas and excel in diverse tasks. This heightened interest and creative prowess contribute to enhanced overall performance (Fredrickson, 2001, as cited in Goodman et al., 2012).

Emotional creativity comprises three distinct levels. The first level pertains to the proficient utilization of one’s emotions. The second level involves the adaptation or modification of one’s emotional responses. The third level encompasses the innovative generation of novel emotional expressions (Averill, 1999, as cited in Goodman et al., 2012). The manifestation of emotional creative response hinges on the interplay of three fundamental criteria: novelty, effectiveness, and originality. Novelty denotes the ability to respond in a manner distinct from one’s customary everyday reactions. Effectiveness encompasses the advantageous outcomes derived by both oneself and others from the emotional response. Originality entails responding in a manner that aligns with one’s values and truthfully expresses one’s authentic self.

## 2. Emotional creativity

Emotional creativity and emotional intelligence share a common thread, yet emotional creativity possesses distinct attributes that bolster individuals in a sustainable manner as they navigate daily emotional reactions. Averill (1980, 2005) contends that emotional creativity can assume both subjective and objective forms, encompassing a diverse array of emotions. Individuals employ various strategies to manage similar situations, highlighting the multifaceted nature of emotional creativity. Emotional creativity is defined as “a pattern of cognitive abilities and personality traits related to originality and appropriateness in emotional experience” (Brito and Fernandes Thomaz, 2022). On the other hand, emotional intelligence pertains to an individual’s capacity to recognize, comprehend, express, regulate, and utilize their own emotions and the emotions of others (Salovey and Mayer, 1990; Bisquerra and Pérez-Escoda, 2007; Peña-Sarrionandia et al., 2015, as cited in Oriol et al., 2016). In recent years, both emotional intelligence (EI) and emotional creativity (EC) have been employed to characterize emotional abilities. Numerous studies underscore the correlation between emotional creativity and behavioral creativity (Ivcevic et al., 2007). Emotional intelligence centers on how individuals reason through emotions, while emotional creativity is intertwined with the depth and intricacy of an individual’s emotional experiences. Consequently, a person with high emotional intelligence might employ diverse regulation strategies, while a person with high emotional creativity is inclined to experience a broader spectrum of complex emotions. Within the context of this study, a key focal point is the trauma symptoms exhibited by undergraduates. As such, measuring emotional creativity proves more advantageous in identifying the intricate range of emotions these individuals navigate amid various traumatic scenarios.

Littrell (2009) established that the expression of emotions is a psychological process, suggesting that the act of expressing emotions can prove beneficial for individuals in a wide array of situations. According to Freud, unresolved conflicts and trauma can trigger emotional responses. If these emotions are not released through expression, they may persist within the body, potentially leading to complications.

Emotional creativity aligns with the concept of emotional intelligence while also maintaining its distinctiveness. Emotional creativity (EC) encompasses the capacity to encounter and convey original, suitable, and genuine amalgamations of emotions (Averill and Thomas-Knowles, 1991, as cited in Martsksvishvili et al., 2017). It encapsulates an array of cognitive abilities and personality traits that contribute to the distinctiveness of emotional aptitude and expression (Zhai et al., 2021).

Novel Emotional reactions can be determined by emotional creativity. The four dimensions of Emotional Creativity are as follows:

Novelty: Acquisition of new knowledge from former behaviors.

Effectiveness: Emotional responses have to be of potential use for the person or group.

Authenticity: Reflection of individual values and beliefs (Authentic nature that is not merely a copy of others’ expectations).

Preparedness: Years of preparation (Averill, 2009, 2013).

Emotional creativity is intertwined with academic motivation, which can be classified into intrinsic motivation and academic engagement (Oriol et al., 2016). Motivation can be categorized into three distinct types: amotivation, extrinsic motivation, and intrinsic motivation (Deci and Ryan, 2002, as cited in Çetin, 2015).

Emotional creativity serves as a resource for individuals seeking to foster their post-traumatic growth (PTG). Particularly in the aftermath of the COVID-19 pandemic, there has been a noticeable increase in the utilization of emotional creativity as a means of development (Zhai et al., 2021).

How does motivation influence our performance within academic settings? Often, motivation acts as a propelling factor behind numerous accomplishments within the academic realm. The theory of achievement motivation primarily centers around three key categories of achievement-oriented motivation. Moreover, alongside these, there exist motivations directed toward affiliation and those driven by the pursuit of power (Kurt, 2021).

The exploration of achievement motivation and its subsequent outcomes has primarily been conducted within the organizational context. Jha (2010) discovered that all motivational needs, except for the need for affiliation, exhibit a notable and positive correlation with psychological empowerment (intrinsic task motivation). Jha’s (2010) study aimed to gauge the impact of motivational needs on psychological empowerment.

### 2.1. Trauma and emotions

As pointed out by Substance Abuse and Mental Health Services Administration Office of Policy (2014), the term ‘trauma’ encompasses experiences that trigger intense physical and psychological stress reactions. It can pertain to a single event, multiple events, or a set of circumstances perceived by an individual as physically and emotionally harmful or threatening, with enduring adverse effects on the individual’s physical, social, emotional, or spiritual well-being.

Traumatic experiences do not exclusively yield negative consequences; rather, they can also engender a capacity for personal growth amidst challenging circumstances. This phenomenon is termed post-traumatic growth (PTG). While major disasters and crisis situations are often linked to adverse psychological effects, they concurrently present an opportunity for growth (Mencius, 2006, as cited in Zhai et al., 2021). The correlation between positive and negative emotions is evident within research findings. Numerous studies have demonstrated a positive correlation between PTG and positive emotions while conversely indicating a negative correlation between negative emotions and PTG.

The social context serves as a milieu wherein individuals navigate a spectrum of emotions and pursue achievements while concurrently encountering potential traumatic experiences. Social support assumes a paramount role within this dynamic framework. Perceived social support encompasses a subjective sensation of being upheld by one’s social network. Notably, individuals endowed with elevated emotional creativity exhibit a heightened capacity to sensitively comprehend the emotions and behaviors of others, thereby adeptly managing emotional conflicts, whether within themselves or in others (Averill and Thomas-Knowles, 1991; Sun and Lu, 2009, as cited in Zhai et al., 2021).

Trauma symptoms, grieving, and other correlated factors stemming from trauma hold substantial importance in comprehending the impact of trauma on an individual’s life (Tay et al., 2016). Emotional facets linked to trauma can be aptly encapsulated through the lens of grief-related factors in trauma. In numerous instances, intensely sorrowful grief responses have the potential to transform into more constructive and positive reactions over time. Depression and physical symptoms are prevalent psychological issues frequently encountered in the aftermath of traumatic experiences (The National Child Traumatic Stress Network, 2018).

Emotions play a pivotal role in the context of traumatic conditions. Individuals afflicted with post-traumatic stress disorder may undergo a reduction in cerebellar volume, thereby impacting the regulation of emotions and attention (Carrion et al., 2013, as cited in Cantrell, 2016).

### 2.2. Research questions

Research Question 1: Can emotional creativity predict lower levels of trauma symptoms among university students?

Research Question 2: Can trauma symptoms predict reduced academic achievements among university students?

Research Question 3: Can emotional creativity predict the degree of academic motivation among university students?

Research Question 4: Does the mediation of trauma symptoms influence achievement motivation resulting from emotional creativity?

### 2.3. Aims and objectives

Teaching in higher educational institutions is a demanding endeavor, necessitating instructors to be attuned to conveying their emotional state distinctly while concurrently accommodating the diverse range of emotional expressions exhibited by students. The primary objective of this study is to delve into the intricate tapestry of emotional experiences stemming from the traumatic events students encounter. Moreover, it seeks to unveil how academic engagement provides a platform for cultivating their emotional wellbeing amidst the backdrop of their trauma. This research primarily aims to discern the influence of emotional creativity and academic motivation in delineating distinct levels of trauma responses among Sri Lankan undergraduates. Furthermore, the study endeavors to uncover the roles of emotional intelligence and achievement motivation in shaping the spectrum of traumatic reactions within the university student population. It also seeks to identify potential variations in emotional creativity levels across different faculties (disciplines). Additionally, the study explores the interplay between emotional creativity and trauma symptoms, the dynamic interaction between achievement motivation and trauma symptoms, and endeavors to pinpoint the impact of emotional creativity on achievement motivation.

### 2.4. Specific objectives

One key objective is to assess students’ levels of emotional creativity across three distinct subscales and their level of academic motivation, with the aim of elucidating the nature of the trauma responses they are grappling with. Another significant goal is to propose a mechanism to the university system for bolstering emotional creativity and implementing programs to enhance achievement motivation. These initiatives aim to assist students in surmounting the challenges posed by traumatic conditions.

### 2.5. Hypothesis

H1. Emotional creativity significantly influences the reduction of trauma symptoms among university students.

H2. Trauma symptoms significantly influence the decrease in achievement motivation among university students.

H3. Emotional creativity impacts the determination of levels of academic motivation among university students.

H4. Emotional creativity is indirectly linked with achievement motivation through the mediation of trauma symptoms.

## 3. Materials and methods

Ethical clearance for conducting this research was granted by the Ethical Review Committee of the Faculty of Medicine, University of Peradeniya, Sri Lanka. Self-report assessments were employed to measure the levels of emotional creativity, academic motivation, and trauma symptoms. To ensure strict adherence to ethical protocols, informed consent documents were meticulously developed to secure participants’ agreement prior to their involvement in the study. The research was carried out at the University of Peradeniya, encompassing a diverse sample from nine distinct faculties, namely, the Faculty of Arts, Faculty of Allied Health Sciences, Faculty of Engineering, Faculty of Agriculture, Faculty of Dental Sciences, Faculty of Medicine, Faculty of Veterinary and Animal Science, and Faculty of Management.

### 3.1. Sample

The sample was chosen through the purposive sampling technique, comprising students from nine distinct faculties at the University of Peradeniya, Sri Lanka. Prior to their participation in the study, participants were provided with informed consent documents, aimed at enhancing their awareness and securing their agreement for the inclusion of data pertaining to them or provided by them in the research. The sample encompassed a total of 377 undergraduates, representing 205 first-year undergraduates, 67 second-year undergraduates, 81 third-year undergraduates, and 24 final-year undergraduates across all faculties at the University of Peradeniya. All participants were provided with a Google Form link, allowing them to complete the questionnaire as part of the self-report assessment. Selection of students was not contingent on specific criteria; any undergraduate from the University of Peradeniya, spanning all nine faculties, was invited to voluntarily partake in the research. The sample selection was unbiased, although a few students chose not to participate, resulting in 337 responses to the questionnaire. Table 2 shows the summary of demographical factors of the sample.

**TABLE 2:** Mean and SD among background information.

	*Min*	*Max*	*M*	*SD*	*St.Sk*	*St.Ku*
Academic year	0	3	0.80	0.988	0.813	−0.705
Faculty	0	8	3.10	2.899	0.468	−1.228
Sex (male/female)	0	1	0.78	0.417	−1.338	−0.212
Religion	0	4	0.50	1.205	2.266	3.503
Monthly Income	0	4	1.42	1.358	0.623	−0.844
Event	0	17	16.05	2.859	−4.029	17.177

M—mean; SD—standard deviation; Min—minimum; Max—maximum; St. Sk—standardized skewness; St. Ku—standardized kurtosis.

### 3.2. Measures

#### 3.2.1. Emotional creativity scale

- Shortened version of Emotional Creativity Inventory (ECI- Averill, 1999).

This scale comprises 30 items designed to assess dispositional personal traits (Averill, 1999) across three dimensions: (1) Preparedness or Emotional Creativity and Academic Engagement emotional disposition, reflecting the capability to comprehend and learn about one’s own and others’ emotions (e.g., “I contemplate my emotions and endeavor to comprehend my emotional reactions”); (2) Novelty, which gauges the capacity to experience novel or unconventional emotions (e.g., “I have encountered an amalgamation of emotions that may be unique to me”); and lastly, (3) Effectiveness/Authenticity, capturing the ability to authentically and effectively express emotions, leading to personal or collective benefits (e.g., “The manner in which I express and live my emotions contributes positively to my relationships with others”). The internal consistency of this scale, as indicated by Cronbach’s alpha coefficient, was 0.91.

#### 3.2.2. McClelland achievement motivation scale

The Achievement Motives Scale (AMS; Man et al., 1994) centers around two fundamental constructs: hope of success and fear of failure. The original iteration of the AMS encompassed 30 items. Subsequently, Lang and Fries (2006) introduced a refined version through confirmatory factor analysis, comprising 150 items. This revised form, known as the Achievement Motives Scale-Revised (AMS-R), has been affirmed to possess satisfactory reliability, diminished inter-scale correlations, and has demonstrated criterion-related validity concerning achievement-related behaviors. The AMS-R is a swift assessment tool that delves into the two closely aligned concepts encapsulated within the framework of achievement motivation theory, rendering it theoretically robust. The internal consistency of this scale, as denoted by the Cronbach’s alpha coefficient, was calculated to be 0.84. As mentioned in the Table 3 there are three subfactors of achievement motivation.

**TABLE 3:** Achievement motivation and sub-factors.

Need for achievement (n ACH)		Need for power (n POW)		Need for affiliation (n AFF)	
Personal responsibility, Feedback, Moderate risk		Influence, competitive		Acceptance and friendship, cooperative	
High	Low	High	Low	High	Low
Must win at any cost, must be on top, and receive credit.	Fears failure, avoids responsibility.	Demands blind loyalty and harmony, does not tolerate disagreement.	Remains aloof, maintains social distance.	Desires control of everyone and everything, exaggerates own position and resources.	Dependent/subordinate, minimizes own position and resources.

Item No 1: I work very hard to continually improve my work performance.

#### 3.2.3. Impact of event scale

The Impact of Event Scale-Revised (IES-R) is a self-report questionnaire consisting of 22 items designed to gauge the subjective distress stemming from traumatic experiences, as per the criteria outlined in DSM-IV (Christianson and Marren, 2008). This revised version builds upon the original 15-item IES (Horowitz et al., 1979), incorporating an additional 7 items dedicated to hyperarousal symptoms of post-traumatic stress disorder (PTSD), which were absent in the earlier iteration. The items directly correspond to 14 of the 17 DSM-IV PTSD symptoms. Respondents are prompted to identify a specific distressing life event and subsequently rate the level of distress or perturbation they experienced over the preceding 7 days in relation to each listed “difficulty.” The internal consistency of this scale, as indicated by Cronbach’s alpha coefficient, was calculated to be 0.78. Furthermore, the translated and back-translated questionnaire underwent rigorous validation through a Delphi process, involving an expert panel. This meticulous validation procedure ensured both the content and concurrent validity of the scale.

Item No 1: Any reminder brought back feelings about it.

## 4. Results

### 4.1. Demographic data

The distribution among faculties was as follows: Faculty of Arts (30.7%), Faculty of Science (5.6%), Faculty of Allied Health Sciences (18.6%), Faculty of Agriculture (14%), Faculty of Dental Sciences (12.4%), Faculty of Medicine (2.1%), Faculty of Engineering (3.6%), Faculty of Veterinary Science (1.9%), and Faculty of Management (11.1%). The total sample consisted of 377 university students from various academic years, encompassing both male and female participants. Among them, 22% were male and 78% were female. In terms of academic years, 54.4% were first-year students, 17.8% were second-year students, 21.5% were third-year students, and 24% were fourth-year students. The sample also exhibited diversity in religious backgrounds, with the distribution as follows: Buddhist (82.5%), Muslim (4.8%), Hindu (1%), Catholic (3.7%), and Other (8%). Regarding the economic background of the students, 32.9% of the sample came from households with a monthly income below Rs. 30,000. Notably, the majority of the participants (83%) indicated experiencing traumatic incidents as “any other stressful event.” Severe human suffering (6.9%) was the next significant incident associated with the development of trauma symptoms. The sudden accidental death of a close individual (5%) emerged as the next frequently reported traumatic experience among the undergraduate students.

### 4.2. Mean and SD of main variables

The traumatic experiences encountered by the students were categorized according to the categories of trauma outlined by the American Psychological Association (APA). Notably, the female gender exhibited a higher prevalence of trauma symptoms. Among both sexes, avoidance emerged as the most frequently reported trauma symptom. Comparing faculties, the level of emotional creativity was notably elevated among students from the Faculty of Arts compared to other faculties. However, across all faculties, emotional preparedness was the least commonly reported component of emotional creativity. On the other hand, emotional authenticity was consistently high among undergraduates across all faculties. The results revealed a prevalent occurrence of trauma symptoms, specifically avoidance (*M* = 14.58, SD = 7.47), intrusion (*M* = 13.85, SD = 7.92), and hyperarousal (*M* = 8.86, SD = 5.64), among undergraduates in all faculties at the University of Peradeniya. Additionally, emotional creativity dimensions were assessed, with emotional preparedness (*M* = 25.5, SD = 3.65), emotional novelty (*M* = 46.32, SD = 8.44), and emotional effectiveness and authenticity (*M* = 30.39, SD = 4.91) being reported. Furthermore, the study measured achievement motivation, with findings indicating that among all undergraduates across faculties, achievement motivation (*M* = 18.84, SD = 2.95), affiliation (*M* = 17.02, SD = 2.81), and power (*M* = 17.14, SD = 2.81) were observed. Comparing faculties, achievement motivation was found to be higher among students from the Faculty of Arts, while it was notably lower among undergraduates in the Faculty of Dental Sciences. It is noteworthy that, despite their faculty affiliations, undergraduates at the University of Peradeniya displayed varying levels of trauma symptoms.

### 4.3. Regression analysis

As summarized in Table 4 a stepwise regression was employed the impact of predictor variables on the outcome variable. The results revealed that two factors within emotional creativity—emotional preparedness and emotional effectiveness and authenticity—had a moderate level of influence on achievement motivation. Additionally, avoidance and hyperarousal emerged as significant trauma symptoms affecting achievement motivation. The regression summary table indicated a relatively low impact of emotional creativity in determining trauma symptoms. Specifically, emotional novelty, emotional effectiveness, emotional authenticity, and emotional preparedness collectively had a lesser influence on the trauma symptom factors of avoidance, hyperarousal, and intrusion. This suggests that the development of emotional creativity may assist individuals in mitigating the subsequent development of trauma symptoms following a traumatic experience. Emotional preparedness exhibited a significant positive influence on achievement motivation (*r* = 0.242, *p* < 0.001, *R*^2^ = 0.217) with a medium effect size. Emotional novelty, however, did not display a significant correlation with achievement motivation (*r* = 0.037, *p* > 0.001). Nonetheless, the impact of emotional novelty was observed collectively with other emotional creativity sub-factors. Emotional effectiveness and authenticity demonstrated a significant positive correlation with achievement motivation among the undergraduate population (*r* = 0.251, *p* < 0.001, *R*^2^ = 0.217). On the other hand, avoidance as a trauma symptom did not display a significant correlation with achievement motivation (*r* = 0.079, *p* > 0.001). However, the impact of this factor was noted alongside the emotional creativity sub-factors in relation to achievement motivation. Intrusion showed a negative correlation with achievement motivation, although it was not statistically significant (*r* = −0.024, *p* > 0.001, *R*^2^ = 0.217). Furthermore, hyperarousal exhibited a significant negative correlation with achievement motivation (*r* = −0.108, *p* < 0.05, *R*^2^ = 0.217).

**TABLE 4:** Summary of regression analysis.

Variables in the regression	Achievement motivation		
	Achievement	Affiliation	Power
Emotional preparedness	0.242***	0.029	0.096
Emotional novelty	0.037	0.046	0.158**
Emotional effectiveness and Authenticity	0.251***	0.258***	0.181***
Avoidance	0.079	−0.066	−0.105*
Intrusion	−0.024	−0.023	0.056
Hyper-arousal	−0.108*	−0.004	−0.005
F	17.06	6.826	11.130
R^2^	0.217	0.100	0.153

*p < 0.05, **p < 0.01, and ***p < 0.001.

Emotional preparedness displayed no significant correlation with affiliation (*r* = 0.029, *p* > 0.001, *R*^2^ = 0.10), with a relatively low impact value. Similarly, emotional novelty exhibited a weak significant correlation with affiliation (*r* = 0.046, *p* < 0.001, *R*^2^ = 0.10), indicating a limited impact on affiliation motivation. Conversely, emotional effectiveness and authenticity revealed a positive and significant correlation with affiliation (*r* = 0.258, *p* < 0.001, *R*^2^ = 0.10), albeit with a minor impact on affiliation motivation. The correlation between avoidance as a trauma symptom and affiliation was negative, yet not statistically significant, and carried a negligible impact value (*r* = −0.066, *p* > 0.001, *R*2 = 0.10). Similarly, intrusion as a trauma symptom exhibited a weak and insignificant correlation with affiliation (*r* = −0.023, *p* > 0.001, *R*^2^ = 0.10). Hyperarousal as a trauma symptom displayed a negative and insubstantial correlation with affiliation (*r* = −0.004, *p* > 0.001, *R*^2^ = 0.10). Regarding the association between emotional preparedness and power as a motivational sub-factor, no significant correlation was observed. However, the impact of emotional preparedness on power was reported to be of moderate magnitude (*r* = 0.096, *p* > 0.001, *R*^2^ = 0.15). Emotional novelty exhibited a noteworthy positive correlation with power as a motivational factor, with a moderate impact (*r* = 0.158, *p* < 0.01, *R*^2^ = 0.15). Similarly, emotional effectiveness and authenticity demonstrated a significant positive correlation with power, also with a moderate impact (*r* = 0.181, *p* < 0.001, *R*^2^ = 0.15). In contrast, avoidance revealed a significant negative correlation with power, exerting a moderate impact (*r* = −0.105, *p* < 0.05, *R*^2^ = 0.15). Intrusion did not exhibit a significant correlation with power; nevertheless, its impact in conjunction with other variables was relatively higher (*r* = 0.056, *p* > 0.001, *R*^2^ = 0.15). Hyperarousal displayed a non-significant negative correlation with power, though its impact, when considered alongside other variables, remained relatively elevated (*r* = −0.005, *p* > 0.001, *R*^2^ = 0.15).

### 4.4. Mediation analysis

Hypothesis 4 (H4), which examined the mediating effect of trauma symptoms on the relationship between emotional creativity and the outcome variable (achievement), was assessed utilizing the Sobel test to ascertain the significance of the mediation pathway. Upon reviewing the results of the mediation analysis, specifically concerning the indirect effect of trauma symptoms on achievement motivation via the mediating role of emotional creativity, an effect size of 0.25 was observed among the undergraduates. The direct effect of emotional creativity on achievement motivation proved to be highly significant (*p* < 0.001), with a notable power of impact (0.419). Conversely, the indirect impact of emotional creativity in mediating the relationship between trauma symptoms and achievement motivation was also found to be significant (*p* < 0.001), with an increased power (Beta = 0.465). This suggests that both the direct and indirect influences of emotional creativity hold high significance. Furthermore, there is a heightened likelihood or increased power associated with utilizing emotional creativity in conjunction with trauma symptoms to predict achievement motivation. In conclusion, the findings emphasize that emotional creativity possesses substantial direct and indirect effects on achievement motivation. Additionally, incorporating the mediating role of trauma symptoms in this relationship enhances the predictive power, thereby highlighting its potential significance in the context of the study. Table 5 shows the summary of mediational analysis using Sobel test.

**TABLE 5:** Summary of the Sobel test analysis.

	Path	Beta (unstand)	SE	Beta (stand)	Significance
Step 1	c	0.207	0.023	0.419	0.001***
Step 2	a	0.550	0.063	0.413	0.001***
Step 3	b	0.030	0.019	0.080	0.119
Step 4	C^1^	0.230	0.25	0.465	0.001***

*p < 0.05, **p < 0.01, and ***p < 0.001.

## 5. Discussion

This study represents a pioneering endeavor within the Sri Lankan context, delving into the interplay between emotional creativity, achievement motivation, and their combined impact on traumatic symptoms.

Our data underscore the role of emotional creativity in mitigating trauma symptoms among university students. Simultaneously, the reported presence of trauma symptoms correlates with diminished aspirations for achievement, as facilitated by achievement motivation. The latter empowers individuals to cultivate skills and behaviors conducive to realizing their full potential. Given the significant commitment of university students to academic pursuits, maintaining a high grade point average (GPA) often leads to elevated stress levels. This combination of academic pressures and traumatic experiences acts as a deterrent to the pursuit of higher achievements.

Among the facets of emotional creativity, emotional effectiveness and authenticity stand out as particularly influential, surpassing other sub-factors. Emotional effectiveness involves appropriate emotional responses aligned with situational context or beneficial outcomes for oneself or a group. Authenticity pertains to genuine expression of personal experiences and values (Ivcevic et al., 2007).

Emotional creativity hinges upon divergent thinking and the generation of original yet fitting responses. While problem-solving is inherent to emotional creativity, experience alone suffices for an emotionally creative response to manifest (Averill, 1999).

Interestingly, emotional creativity exhibits variations among faculties, with the Faculty of Arts displaying heightened levels. This discrepancy can be attributed to the creative tasks required within Arts programs and the competitive career paths chosen by Arts students. Notably, the link between emotional creativity and achievement motivation is corroborated, aligning with Oriol et al.’s (2016) findings.

The impact of emotional creativity and trauma symptoms on achievement motivation extends to its components, where achievement and power hold significant sway. Emotional novelty and emotional effectiveness and authenticity positively correlate with power, reflecting their contribution to autonomous decision-making.

Among the predictor variables, emotional effectiveness and authenticity emerge as the most influential. Both achievement motivation and power are shaped by these variables, although the influence is of medium magnitude. Importantly, the combined influence of emotional creativity and trauma symptoms on achievement levels surpasses that of all other sub-factors.

Trauma symptoms are found to decrease achievement motivation, as indicated by the regression analysis. Notably, emotional preparedness and emotional novelty wield substantial impact in this context, with novel emotions enhancing one’s autonomy in new situations.

Faculty affiliation is also affected by emotional creativity, particularly emotional effectiveness and authenticity. Authentic emotional expression bolsters affiliations within the academic sphere.

In conclusion, developing emotional creativity can serve as a strategic approach for students to counteract the mediating effect of trauma symptoms on achievement motivation. The mediation analysis affirms the potency of emotional creativity in this interplay.

Several limitations are acknowledged in this study. Future research could integrate qualitative interviews alongside quantitative methods. Additionally, delving into students’ coping mechanisms for traumatic events and exploring factors such as culture, media influence, and the globalization transition could enrich our understanding. Notably, in Sri Lanka, the disruptions caused by the COVID-19 pandemic and economic recession demand flexible and culturally sensitive coping strategies for traumatic experiences among university students.

## 6. Conclusion

Research Question 1 aimed to ascertain the predictability of emotional creativity on trauma symptoms among university students. The results demonstrated that a higher level of emotional creativity corresponds to a reduction in the experienced magnitude of trauma symptoms in university students. Research Question 2 explored the predictability of trauma symptoms on achievement motivation. The findings highlighted that the presence of trauma symptoms is associated with diminished levels of achievement among university students. Research Question 3 delved into the impact of emotional creativity on achievement motivation. The influence of emotional creativity on achievement motivation was found to be of medium magnitude. Research Question 4 centered on the potential mediation effect of trauma symptoms in determining achievement motivation through emotional creativity. The mediation analysis yielded the conclusion that both the direct and indirect effects of emotional creativity hold significant sway. Notably, the emergence of emotional creativity can be influenced by trauma symptoms. Among the facets of emotional creativity, emotional preparedness and emotional effectiveness and authenticity emerged as particularly influential factors. Future research avenues could embrace a mixed-method approach, where quantitative and qualitative methods synergize to explore other potential factors influencing achievement motivation amidst traumatic experiences.

## Data availability statement

The original contributions presented in this study are included in the article/supplementary material, further inquiries can be directed to the corresponding author.

## Author contributions

AB conceived the study design, contributed to the conclusion, and drafted the manuscript. AB and RL contributed to the discussion, recruited and collected data, analyzed data, interpretation of the results, and approved the submitted version.
